# The frail patient undergoing cardiac surgery: lessons learned and future perspectives

**DOI:** 10.3389/fcvm.2023.1295108

**Published:** 2023-12-06

**Authors:** Matteo Pozzi, Silvia Mariani, Margherita Scanziani, Davide Passolunghi, Adriana Bruni, Alberto Finazzi, Maddalena Lettino, Giuseppe Foti, Giuseppe Bellelli, Giovanni Marchetto

**Affiliations:** ^1^ Department of Emergency and Intensive Care, IRCCS San Gerardo dei Tintori Foundation, Monza, Italy; ^2^ Cardiovascular Research Institute Maastricht (CARIM), Maastricht, Netherlands; ^3^ Division of Cardiac Surgery, IRCCS San Gerardo dei Tintori Foundation, Monza, Italy; ^4^ Acute Geriatrics Unit, IRCCS San Gerardo dei Tintori Foundation, Monza, Italy; ^5^ School of Medicine, University of Milan Bicocca, Monza, Italy; ^6^ Department of Cardiovascular Medicine, IRCCS San Gerardo dei Tintori Foundation, Monza, Italy

**Keywords:** frailty, cardiac surgery, minimally invasive cardiac surgery, ERAS, comprehensive geriatric assessment

## Abstract

Frailty is a geriatric condition characterized by the reduction of the individual's homeostatic reserves. It determines an increased vulnerability to endogenous and exogenous stressors and can lead to poor outcomes. It is an emerging concept in perioperative medicine, since an increasing number of patients undergoing surgical interventions are older and the traditional models of care seem to be inadequate to satisfy these patients' emerging clinical needs. Nowadays, the progressive technical and clinical improvements allow to offer cardiac operations to an older, sicker and frail population. For these reasons, a multidisciplinary team involving cardiac surgeons, clinical cardiologists, anesthesiologists, and geriatricians, is often needed to assess, select and provide tailored care to these high-risk frail patients to optimize clinical outcomes. There is unanimous agreement that frailty assessment may capture the individual's biological decline and the heterogeneity in risk profile for poor health-related outcomes among people of the same age. However, since commonly used preoperative scores for cardiac surgery fail to capture frailty, a specific preoperative assessment with dedicated tools is warranted to correctly recognize, measure and quantify frailty in these patients. On the contrary, pre-operative and post-operative interventions can reduce the risk of complications and support patient recovery promoting surgical resilience. Minimally invasive cardiac procedures aim to reduce surgical trauma and may be associated with better clinical outcome in this specific sub-group of high-risk patients. Among postoperative adverse events, the occurrence of delirium represents a risk factor for several unfavorable outcomes including mortality and subsequent cognitive decline. Its presence should be carefully recognized, triggering an adequate, evidence based, treatment. There is evidence, from several cross-section and longitudinal studies, that frailty and delirium may frequently overlap, with frailty serving both as a predisposing factor and as an outcome of delirium and delirium being a marker of a latent condition of frailty. In conclusion, frail patients are at increased risk to experience poor outcome after cardiac surgery. A multidisciplinary approach aimed to recognize more vulnerable individuals, optimize pre-operative conditions, reduce surgical invasivity and improve post-operative recovery is required to obtain optimal long-term outcome.

## 1. Clinical frailty: definition and pathophysiology

From the latin “fragilis” meaning “easily broken”, frailty is a geriatric syndrome defined as a form of vulnerability to stress due to decline of physiologic reserve (1). In particular, frailty is a multidimensional condition involving many organ systems, general health status, physical and cognitive functions, nutritional state, skeletal muscle mass, strength and mobility, mood, social support and relations (2). Although aging is closely linked to frailty, and frailty is often seen in older adults, frailty can be present also in younger people (3). This condition is clinically characterized by the presence of some key signs, such as weakness, slow gait speed, poor mobility, fatigue and unintentional weight loss (2).

From a pathophysiological point of view, a two-way relationship between cardiovascular diseases (CVD) and frailty has been proposed (4). According to the current hypothesis, a proinflammatory state occurring with aging represents the key factor of the phenotypic modifications observed in frail subjects, leading to cellular damage, catabolic muscles modifications, impaired homeostasis and ultimately vulnerability to external stressors (5). As CVD shares a common etiological pathway, these two conditions are clinically and epidemiologically closely linked with a significant amount of frail persons among CVD patients (6, 7).

## 2. Frailty and surgery

Surgery can be considered a major stressor able to unveil a silent frailty condition or to dramatically decompensate an overtly frail patient. Since frail subjects are increasingly represented among surgical patients, their identification in the perioperative phase has become crucial. This has prompted careful preoperative selection of cases, appropriate management of pre-operative and post-operative conditions, and adequate estimation of long-term outcomes (8).

The association between frailty and adverse postoperative outcome in adult non-cardiac surgery patients has been extensively described. In particular, frail patients are at higher risk to develop postoperative delirium (9), cardiovascular events (10), and procedural complications (10–13). They are characterized by a slower recovery (14, 15), prolonged intensive care unit (ICU) and in-hospital stay (14, 16) and ultimately morbidity and mortality (10, 17), with a huge increase in global medical costs (16, 18). Moreover, this association between frailty and postoperative adverse outcomes seems independent from patients' age, comorbidities and the procedural-related surgical risk. A recent analysis of a large US database revealed that among patients undergoing non-cardiac surgery, those with higher frailty risk score (19) have higher risk for perioperative cardiovascular events and mortality. They require more frequent discharge to short–term acute care or intermediate care facilities compared to those with lower frailty, in all age groups and independently from patients comorbidities (20). Moreover, in a large longitudinal cohort study, the independent association of frailty with an increased postoperative mortality was retained not only in high risk procedures but also in low-risk procedures from low-intensity surgical specialties (21).

The increasing number of aging patients living with more comorbidities, together with the improvement of surgical outcome in the older population, led to a large proportion of frail patients in cardiac surgery. However, cardiac surgery is associated with a high degree of invasiveness and iatrogenic stress that can compromise postoperative outcomes in frail patients with reduced ability to face such distress. As the aim of each cardiac operation is to restore biological integrity and functional capacity so as to improve the patient's quality of life (QoL), the inability to face the surgical stress may ultimately compromise the net result of a surgical procedure. Indeed, frailty was reported to be an independent predictor of in-hospital and mid-term mortality in a large Canadian cardiac surgery population (22). These data were confirmed by a meta-analysis including more than 60,000 patients (23). As in non-cardiac surgery patients, this association is maintained independently of age (24) or surgical risk score (25) and is proportional to the degree of frailty (23, 24). Moreover, pre-operative vulnerability is not only associated with major postoperative complications and prolonged hospitalization (25), but also with worse post-discharge QoL up to one year after surgery (26).

**Figure 1:**
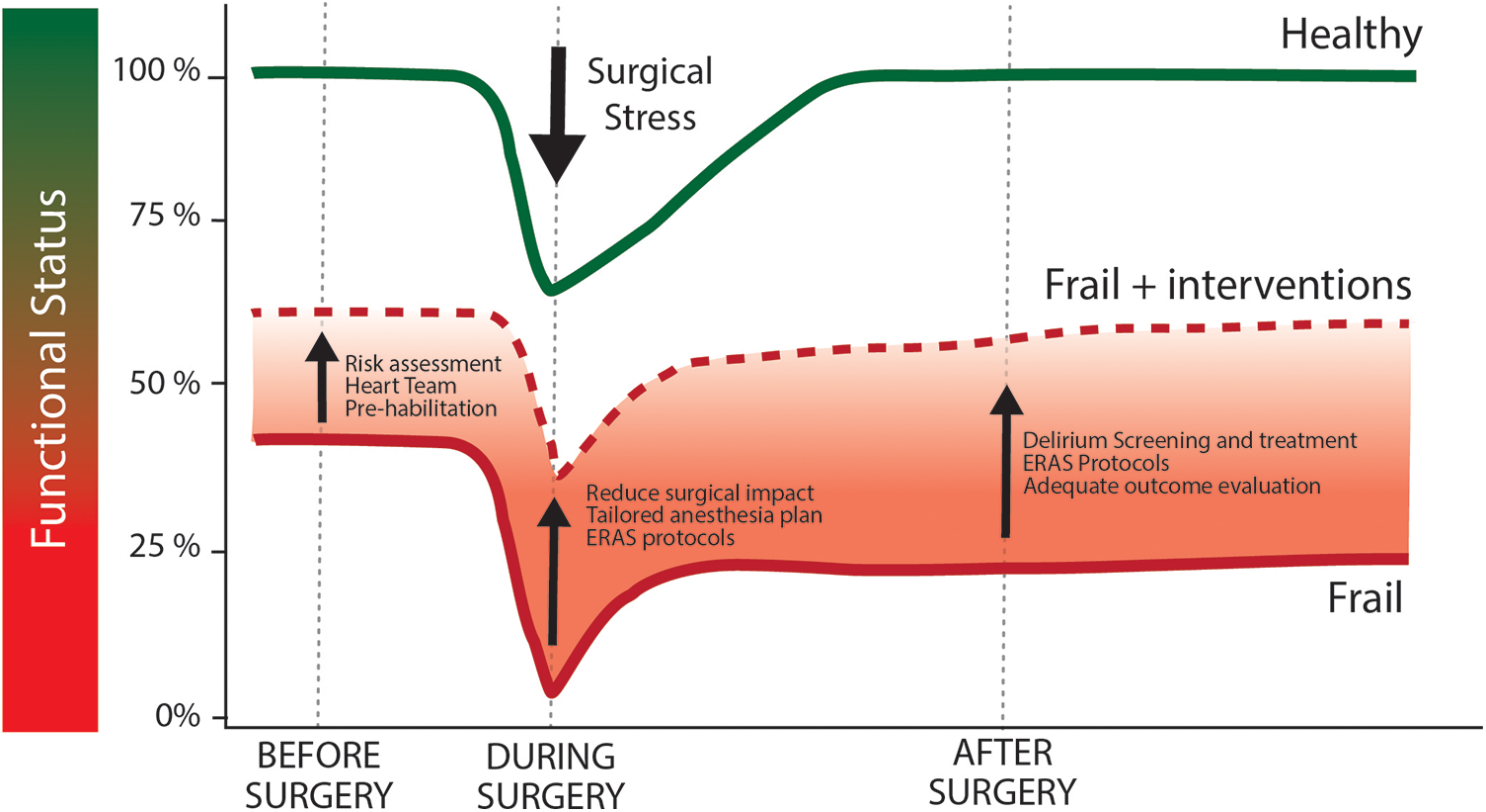
Effect of surgical stress and optimization measures above functional status trajectories in healthy and frail patients. Following the stress of cardiac surgery healthy individuals (green line) suffer from a acute worsening of functional status, that promptly return to baseline during post-operative period. Frail individuals (red line) are characterized by a compromised baseline functional capacity. Similarly to healthy individuals, functional capacity is further reduced after surgery. As frail patients are not able to face such stressing factor, they could not be able to return to baseline functional capacity during post-operative period. This circumstance can compromise long-term outcome and ultimately the net result of the surgical procedure. Functional capacity trajectory could be modified by pre-operative, intra-operative and post-operative specific intervention (dashed red line), whose aim is to improve baseline conditions and to reduce the stressful burden of cardiac surgery.

For the above mentioned reasons, it can be postulated that the active pre-operative recognition of frail patients may help to ameliorate their own outcome and impact patient management in different moments of the clinical course from risk stratification to pre-habilitation programs and surgical choices (Figure 1). The first step to improve outcomes in frail patients is the recognition of their condition at the moment of surgical indication. Indeed, its recognition allows clinicians to formulate a more precise risk estimation (27) based on a precise and commonly accepted definition of frailty. This issue has important consequences on the communication with patients and families, as it involves the shared definition of the goals of care, ensures a patient-centered treatment and avoids disproportionate treatments or futility. The second step implies the reduction of patients' vulnerability by means of a “pre-habilitation” program (28) involving physical, respiratory and nutritional preoperative optimization. Last, identification of more vulnerable patients could promote the tailoring of the best perioperative pathway for each patient, in particular in terms of minimally invasive surgical options and postoperative care bundles.

Such a holistic clinical approach matches up well with the already conceptualized organizational model of the “Heart Team” (28), which includes the participation of physicians from different disciplines (e.g., cardiac anesthesiologists, geriatricians, internal medicine physicians) in addition to cardiologists and cardiac surgeons to provide the best comprehensive management of cardiac conditions in frail patients. The following paragraphs will systematically investigate the available tools to optimize the clinical course of frail patients in cardiac surgery.

## 3. Pre-operative assessment of frail patients

### 3.1. Predictive scores in cardiac surgery

Estimation of surgical risk relies upon the use of scoring systems to predict patients' risk of adverse outcomes. Traditionally, two main risk scoring systems have been available in cardiac surgery: the Society of Thoracic Surgery (STS) Predicted Risk of Mortality or Major Morbidity (29) and the EuroSCORE (30).

Both these scores incorporate age, major comorbidities and traditional physiological variables, but they do not consider variables such as liver cirrhosis, right ventricle function and frailty (31) which are becoming frequent among cardiac patients. Consequently, these scores lost their predictive performance. If STS Score tends to underestimate the risk in more vulnerable patients (32), the old versions of EuroSCORE were burdened by a systematic overestimation of perioperative risk. Although they have been recalibrated (30, 33) high-risk patients remain excluded from accurate risk predictions. In particular the new EuroSCORE II partially reduces the overprediction of the previous versions, at the cost of a tendency toward miscalibration in high-risk groups (31, 32, 34).

Moreover, the inclusion of risk factors for mortality that are very rare in surgical population but with a dramatic impact on the outcome poses some unresolved statistical issues. As an example, advanced liver cirrhosis in extremely rare in surgical population (<0.5%), but mortality associated with this condition is probably more than 70% (35).

Already in 2010, it has been described that slow gait speed, a clinical marker for frailty, confers a 2- to 3- fold increase in risk for any given level of STS predicted mortality and major morbidity (27). Based on the growing evidence that the addition of variables related to frailty could improve the predictive power of these scoring systems (27), frailty was partially incorporated into the available scores. Consequently, STS score version 2.73 included gait speed as a marker of patient frailty, while in the revised version of EuroSCORE II the variable “neurologic dysfunction” was replaced by “poor mobility”, as a generic component of frailty phenotype (30). Although this can be considered a first important step, these scores will continue to underestimate the impact of frailty on patient's outcomes. Moreover, gait speed evaluation is often not performed in the daily routine of cardiac surgery patients (36).

### 3.2. Beyond eyeball evaluation: how to measure frailty using the comprehensive geriatric assessment

Besides the traditional risk scores for cardiac surgery patients, specific tools have been designed to test frailty. In 2001, Fried and colleagues introduced the concept of a physical phenotype model to clinically characterize frailty (2). According to this model, older adults can be diagnosed with frailty if they exhibit three or more out of five criteria: unintentional weight loss of ≥10 pounds in the last year, weakness (determined by grip-strength), exhaustion, low physical activity, and slowed walking speed (2). Similarly, Rockwood and Mitnitski proposed a frailty index (FI), based on an accumulation of age-related deficits model (37). In their model, frailty is quantified as a continuous score that sums up signs, symptoms, disabilities, and diseases (37).

Frailty leads to various clinical consequences and manifestations, including cognitive impairment, loss of independence in daily activities, reduced mobility, and even mortality. Regardless of the specific tool employed, the diagnosis of frailty can be attained by gathering information about an individual's physical performance, mobility, cognitive and nutritional status. In this context, the comprehensive geriatric assessment (CGA) emerges as a robust method to capture frailty's essence (38). CGA involves administering specific scales to assess comorbidities and number of medications, functional ability, nutritional status, mobility, cognition and mood, physical activity and risk of falls, and socioeconomic status (Table 1). Hereafter the most significant domains for frailty assessment are presented.

**Table 1:** Domains of the comprehensive geriatric Assessment and corresponding instrument for clinical assessment.

Domain	Topic investigated	Clinical Assessment instruments
Health status	Chronic diseases, multimorbidity and polypharmacy	• Charlson Index • Cumulative Illness Rating Scale • Number of medications
Nutritional status and sarcopenia	Malnutrition	• Mini Nutritional Assessment • Geriatric Nutritional Risk Index • Serum albumin levels • Anthropometric measures handgrip strength • Bioelectrical impedance analysis
Cognition and mood	Cognitive status and affective disorders	• Mini Mental State Examination • Montreal Cognitive Assessment • Geriatric Depression Scale
Functional status	Basal and instrumental activities of daily living	• Activities of Daily Living • Instrumental Activities of Daily Living
Mobility	Gait and balance	• Gait speed • Chair Stand Test • Short Physical Performance Battery
Socioeconomic status and quality of life	Home care, long term care service, nursing homes, income	• Cohabitation status • Short Form Health Survey 36

#### 3.2.1. Health status

Health status encompasses medical history, multimorbidity and polypharmacy. The Cumulative Illness Rating Scale (CIRS) is a comprehensive tool used to assess an individual's overall health status by evaluating the presence and severity of various medical conditions across different body systems. The CIRS aims to provide a holistic picture of an individual's health by considering the cumulative impact of multiple medical conditions on the well-being. The scale is widely used in clinical and research settings to assess the overall health and functional capacity of individuals, particularly in the context of aging and chronic diseases (39). The Charlson Comorbidity Index (CCI) is a widely used scoring system that quantifies the burden of comorbidities or underlying medical conditions in a patient and their potential impact on mortality (40). Each condition is assigned a weight, and these weights are summed to calculate an overall score for an individual patient. The higher the score, the greater the burden of comorbidities. The CCI is commonly used in clinical research and healthcare settings to assess the overall health status and to predict the risk of mortality or other adverse outcomes. It provides a standardized way to account for the presence and severity of comorbidities. The index has been validated and adapted for various medical conditions and populations.

#### 3.2.2. Functional status

Functional status refers to an individual's ability to perform daily activities necessary for independent living and self-care. Functional status is often categorized into two main components: Activities of Daily Living (ADLs) and Instrumental Activities of Daily Living (IADLs). The ADLs encompass the fundamental self-care activities that are essential for maintaining one's personal well-being and functioning daily, including bathing, dressing, toileting, transferring, continence and eating (41). The Instrumental Activities of Daily Living (IADLs) include 8 complex activities related to the ability to live independently in the community, such as managing finances and medications, meal preparation, housekeeping, laundry, transportation, communication, and shopping (42).

#### 3.2.3. Mobility impairment

Mobility impairment in older individuals can have a significant impact on their health status. The ability to move and navigate one's environment is crucial for maintaining independence, participating in daily activities, and enjoying a high QoL. Gait speed is a simple test to assess mobility in older adults. This single-item test involves timing individuals while they walk at a steady pace for a set distance, usually 4 meters. Generally, a gait speed greater than 5 s for 4 meters (<0.85 m/s) is associated with an increased risk of having frailty (43). The Short Physical Performance Battery (SPPB) (44) is a tool designed to evaluate the physical functioning and mobility of older adults. It is helpful in identifying age-related declines in physical performance and predicting functional limitations. The SPPB consists of a series of three tests (balance tests, gait speed test and chair stand test) that together provide a comprehensive picture of an individual's lower extremity function and overall physical capacity. The tests are simple, quick to administer, and require minimal equipment.

#### 3.2.4. Cognitive functions and mood disorders

The assessment of cognitive function is a crucial component of CGA. Utilizing validated screening tools like the Mini-Mental State Examination (MMSE) (45) and Montreal Cognitive Assessment (MOCA) (46), physicians can quickly evaluate an individual's cognitive abilities, including memory, attention, language, and executive function. Identification of cognitive deficits enables timely intervention, such as cognitive rehabilitation and targeted support, to mitigate functional decline and enhance overall QoL. Moreover, the Geriatric Depression Scale (GDS) aids in detecting depressive symptoms (47), which can intersect with cognitive impairment. By addressing cognitive well-being, the CGA contributes to a holistic understanding of a frail person's health and guides tailored care strategies.

#### 3.2.5. Nutritional status

Adequate nutritional status is essential for maintaining physical health, supporting immune function, and preventing chronic diseases. Measuring nutritional status involves assessing various factors related to an individual's diet, body composition, and overall health. The Mini Nutritional Assessment (MNA) (48) and the Geriatric Nutritional Risk Index (GNRI) (49) are instruments to explore nutritional status of the elderly. The MNA is composed of simple measurements and brief questions that can be completed in about 10 min. The sum of the MNA score distinguishes between older people with adequate nutritional status, protein-calorie malnutrition or at risk of malnutrition. The Geriatric Nutritional Risk Index (GNRI) (49) is an objective and easy screening method based on height, weight, and serum albumin level. Additional evaluations to assess nutritional status include anthropometric measurements, such as Body Mass Index (BMI), blood biomarkers, such as albumin serum levels and body composition analysis, such as bioelectrical impedance analysis (BIA).

#### 3.2.6. Hand grip strength

Hand Grip Strength (HGS) measurement assess muscle function and overall health in older adults (50). It provides a reliable measure of muscle strength, an indirect measure of functional independence and can predict the risk of fall and other adverse outcomes. HGS can be measured using a handheld dynamometer. Three trials for each hand are performed, and the highest value of the strongest hand is recorded. BMI-adjusted values are used to identify low muscle strength in females and males.

#### 3.2.7. Polypharmacy

Polypharmacy refers to the simultaneous use of multiple medications by an individual, typically involving the use of five or more different medications. These medications can include prescription drugs, over-the-counter medications, and even herbal or complementary remedies. Polypharmacy becomes particularly relevant in older adults with multiple chronic diseases and various medications to manage their health (51). Polypharmacy is a significant issue due to its potential to cause adverse drug reactions, decrease medication adherence, and negatively impact the overall health and well-being of older adults. As a result, healthcare professionals specializing in geriatrics must carefully assess and manage medication regimens to ensure that the benefits of each medication outweigh the risks, and to promote optimal health outcomes for their older frail patients.

#### 3.2.8. Socioeconomic status and quality of life

Socio-Economic Status (SES) plays an important role since a high SES provides older adults with material resources, helps them develop healthy lifestyles, and confers psychological benefits. Consequently, older adults with a higher SES tend to have a lower likelihood of mortality than their lower SES counterparts (52). The Short Form Health Survey 36 (SF-36) is validated for the assessment of QoL, the questionnaire consists of 36 questions covering 8 domains (physical functioning, role-physical, bodily pain, general health, vitality, social functioning, role-emotional and mental health), scaled from 0 to 100, a higher score indicates a better QoL. The domains are summarized into physical and mental health scores (53).

In summary, CGA is the most reliable approach to identifying the frail patient and to making a personalized care plan. However, it is not always possible to take a complete evaluation given the different settings and available resources. Therefore, health care professionals need a frailty assessment that is simple, not time-consuming, and helpful in making decisions about interventions and care allocation. The Essential Frailty Toolset (EFT), for example, has shown to be easy to use and predictive of adverse events in patients undergoing aortic valve replacement procedures (54) and coronary artery bypass grafting in older adults (55). The EFT is scored 0 (least frail) to 5 (most frail) based on the following 4 items: pre-procedural anemia, hypoalbuminemia, lower-extremity muscle weakness defined as a time of ≥15 s or inability to complete five sit-to-stand repetitions without using arms, and cognitive impairment defined as a score of <24 on the Mini-Mental State Examination. Although the EFT is not all-encompassing, it is a well-rooted starting point to test for frailty, and to identify patients in whom further geriatric assessment should be considered to confirm the diagnosis of sarcopenia, malnutrition, dementia, depression, or disability.

### 3.3. Incorporating frailty evaluation into clinical practice: the Heart Team model

Starting from the belief that a multidisciplinary approach is frequently required to manage and ensure better care for patients, international recommendations progressively emphasized the importance of the Heart Team (HT) in all fields of cardiology and cardiac surgery. Indeed, current guidelines strongly recommend HT implementation for optimal management of valvular disease (56), heart failure (57) and myocardial revascularization (58). Nowadays, in addition to cardiologists and cardiac surgeons, HT includes heart imaging specialists, anesthesiologists, ICU physicians and other specialists (e.g., neurologist, nephrologist, geriatrician) whose contribution is required by the specific patient condition. As comprehensive frailty evaluation improves perioperative risk prediction (27), and since a huge amount of patients with CVD are deemed to be frail (6, 7), geriatricians play an increasingly important role in this multidisciplinary patients management.

HT physicians are committed to a unique purpose: to provide a precise risk stratification of the patient and then to identify the best treatment strategy. Such a treatment plan cannot disregard patients' own wishes as shared decision-making improves surgical outcomes and QoL. In this way, the role of HT is to holistically put the patients' complexity at the center of medical decision-making.

Besides perioperative risk quantification and frailty evaluation, treatment decisions should take into account life expectancy. Statistical estimation of the averaged remaining years of life at a single patient level is not a simple task, in particular in elderly patients with multiple associate conditions, but different scores have been developed for this purpose (59). In this perspective, this evaluation can help to balance perioperative short term mortality and morbidity risk with long term survival expectancy (60).

According to the guidelines of the European Society of Cardiology (ESC) and the European Society of Cardio-Thoracic Surgery for the management of valve diseases, frailty assessment should always precede the final decision concerning the type of chosen intervention and its timing, particularly in elderly patients (56). For example, in the flowchart that outlines the management of patients with severe aortic stenosis comorbidity and frailty assessments are mandatory to decide whether any kind of intervention is likely to be of benefit and should be considered during the HT decision process. Similarly to the ESC recommendation also The American College of Cardiology and American Heart Association emphasize shared decision-making in cardiac surgery, taking into account patients' values, preferences, and frailty status (61). Both suggest the use of validated frailty scores such as the Katz index (41) to grade the level of frailty and take operative decisions accordingly.

Despite preoperative frailty assessment and prehabilitation practices have been recommended by both the aforementioned guidelines and several consensus documents, such as the ones issued by the Society of Perioperative Assessment and Quality Improvement (SPAQI) (62) and the Enhanced Recovery After Surgery (ERAS) Society (63), frailty is so far not being routinely assessed before surgery for many different reasons. First of all, neither a comprehensive geriatric assessment nor any intervention to optimize the patient's condition and reduce complications are feasible under acute conditions. In addition, many clinical tools to assess frailty require patient's active participation and this is not always the case for patients with a poor clinical, social or educational status (64). Finally, there is still a lack of consensus among different frailty instruments that might affect anesthesiologists' and surgeons' behavior, suggesting the opportunity to develop a more practicable and validated workflow in this specific context.

## 4. Pre-operative optimization: *is frailty a modifiable factor?*

As previously mentioned, the preoperative identification of frail patients can trigger the development of dedicated programs to improve the preoperative patients' condition. In this context, the Enhanced Recovery After Surgery (ERAS) guidelines has gained popularity and are increasingly applied (63). ERAS is a multimodal, multidisciplinary care improvement initiative to promote recovery of patients undergoing surgery throughout their entire perioperative journey (65). Modifiable factors addressed by the ERAS recommendations include an optimal perioperative glycaemic control, defined by a hemoglobinA1c level less than 7% (66), and an evaluation of hypoalbuminemia (63). For patients who are malnourished or have a serum albumin level less than 3.0 g/dl, nutritional supplementation for 7 to 10 days before surgery may improve outcomes (63, 67). Carbohydrate loading shortly before surgery might be considered to improve postoperative glucose control and gut function but evidence to support the routine application of this strategy are still lacking (63). Intake of clear liquids until 2 to 4 h preoperatively may be considered before general anesthesia but further studies are required to investigate the risk of aspiration pneumonitis in cardiac surgery patients undergoing intraoperative transoesophageal echocardiography or characterized by delayed gastric emptying due to diabetes mellitus (63). Screening for excessive alcohol use and cigarette smoking should be performed (68) and consumption should be stopped 4 weeks before elective surgery (69).

### 4.1. Patient engagement and prehabilitation

Pre-operative assessment of fragile patients leads to the application of preventive interventions (prehabilitation) including inspiratory muscle training, functional exercise training, psychological support (anxiety and depression reduction), nutritional support, and smoking cessation (63, 70). It has been demonstrated that such strategies, together with the optimization of modifiable factors, may reduce the length of hospital stay, decrease the postoperative morbidity (especially in terms of pulmonary complications) and mortality, and improve the transition from the hospital to the community (71–73). Table 2 provides a summary of the previous randomized studies evaluating the effect of prehabilitation protocols on different perioperative outcomes.

**Table 2:** Available randomized trials evaluating prehabilitation.

Paper	Study design	Sample Size	Type of Surgery	Intervention	Main Results
Arthur et al. (74)	RCT	246	CABG	Multimodal prehabilitation before planned cardiac surgery. Outpatient setting	Reduction of postoperative ICU LOS (by 2.1 h, 95% CI -1.2–16 h, *p* = 0.001) and Hospital LOS (by 1 day, 95% CI 0–1, *p* = 0.002). Better preoperative and postoperative quality of life. No differences in mortality.
Herdy et al. (75)	RCT	56	CABG	Multimodal Prehabilitation before planned cardiac surgery. Hospitalized patients.	Shorter duration of mechanical ventilation. Reduction of pleural effusion (RR = 0.2; 95% CI: 0.5–0.8), atelectasis (RR = 0.15; 95% CI: 0.03–0.8), and AF (RR = 0.2; 95% CI: 0.05–0.8). Reduction of in-hospital LOS (5.9 ±/−1.1 vs. 10.3 ±/−4.6 days, *p* < 0.001).
Rosenfeldt et al. (76)	RCT	117	CABG, valve surgery	Multimodal Prehabilitation before planned cardiac surgery. Outpatients setting	No differences in quality of life, LOS and Atrial Fibrillation.

RCT, Randomized Clinical Trial; CABG, Coronary Artery Bypass Graft; ICU, Intensive Care Unit; LOS, Length of Stay; CI, Confidence Interval; AF, Atrial Fibrillation; RR, Relative Risk.

Patient education and counseling prior to surgery can be completed in person, through printed material, or through application-based approaches (63). As telemedicine has become widely adopted, especially during the COVID-19 pandemic (77, 78), personalized prehabilitation programs may also be delivered using this technology, as already described in the field of cardiac rehabilitation (79, 80).

## 5. Intraoperative choices for frail patients: the right strategy for the right patient

Besides the preoperative targeting of modifiable factors and prehabilitation programs, much can be done intraoperatively to optimize patients' outcomes. A multidisciplinary approach should guide the intraoperative management in terms of surgical technique and anaesthesiologic strategies, with careful preoperative planning. It has been demonstrated that with a proper preoperative patient assessment in terms of past medical history, comorbidities, and anatomy and with an adequate allocation to the most appropriate surgical and anaesthesiologic approach, the overall rate of early mortality and main complications remain low (81). Indeed, the era of the “one size fits all” approach in cardiac surgery has now been overtaken by precision medicine and tailored surgery. New technologies such as virtual reality (82) and 3D printing (83) can further assist in surgical planning. Hereafter the different options for surgical approach according to different cardiac conditions are presented (Table 3).

**Table 3:** Surgical approaches in frail patients.

Disease	Surgical principles in frail patients	Available techniques
Coronary artery disease	Reduce aortic manipulation Minimize surgical incision Consider LITA-LAD + PCI of other vessels	Anaortic coronary artery bypass: off-pump + no touch Left thoracotomy: MIDCAB, MICS CABG Robotic surgery: TECAB Hybrid revascularization
Aortic valve disease	Minimize surgical incision Reduce CPB time	Mini-sternotomy / Mini-thoracotomy /Right Mini-thoracotomy Sutureless and rapid deployment valves
Mitral valve disease	Minimize surgical incision Consider trans-apical off-pump approaches	Right Mini-thoracotomy NeoChord Trans-catheter valves
Tricuspid valve disease	Minimize surgical incision Reduce CPB time	Right Mini-thoracotomy Beating heart right heart surgery
Ascending aorta and aortic arch	Minimize surgical incision Reduce cardiac ischemic time Consider debranching of supra-aortic vessels + EVAR Consider total endoscopic approach	Mini-sternotomy Beating heart aortic surgery Hybrid arch repair Fenestrated/branched arch endografts
Heart failure	Minimize surgical incision	LVAD implantation with right thoracotomy + mini-sternotomy

CABG, coronary artery bypass graft; CPB, cardiopulmonary bypass time; EVAR, endovascular aneurysm repair; LAD, left anterior descending artery; LITA, left internal thoracic artery; LVAD, left ventricular assist device; MICS, minimally invasive cardiac surgery; MIDCAB, minimally invasive direct coronary artery bypass; PCI, percutaneous coronary intervention; TECAB, total endoscopic coronary artery bypass.

### 5.1. Coronary artery bypass graft (CABG)

CABG surgery remains the most frequently performed operation in adults. Nevertheless, cardiopulmonary bypass and aortic cross-clamping may be related to complications in fragile patients with porcelain ascending aorta and/or atheroma in the ascending aorta or arch. These patients might suffer from embolization due to the aortic cross-clamp, the jet from the aortic cannula inflow and, in the case of a porcelain aorta, aortic rupture and or dissection from a cross-clamp injury. A simple preoperative screening with a non-contrast computed tomography or an intraoperative epiaortic scan helps in the triage of these patients (Class IIa indication in the EACTS/ECC 2018 Coronary Revascularization Guidelines) to the appropriate surgical technique including no-touch approaches or hybrid minimally invasive approaches (58).

Anaortic CABG is a technique of off-pump coronary artery revascularization that avoids aortic manipulation by often using all arterial grafts. Typically, the mammary arteries are used for in-flow and the radial artery as a composite graft (84). This technique is particularly indicated for patients with a diseased ascending aorta (Class I indication in the EACTS/ECC 2018 Coronary Revascularization Guidelines and Class 2a indication in the most recent AHA coronary guidelines) (58, 85).

Where expertise exists, minimally invasive direct CABG (MIDCAB) through limited thoracic access should be considered in patients with isolated lesions on the left anterior descending artery (LAD) or in the context of hybrid revascularization strategies (Class IIa indication in the EACTS/ECC 2018 Coronary Revascularization Guidelines) (58). The MIDCAB operation is characterized by LAD grafting with the left internal thoracic artery through a left anterior small thoracotomy. It can be combined with percutaneous coronary intervention (PCI) of non-LAD coronary stenoses in a sequential or concomitant way. This latter approach is defined as hybrid revascularization where stents become substituted for saphenous vein grafts for non-LAD lesions (86).

Minimally invasive coronary surgery (MICS CABG) was developed as an extension of the MIDCAB operation and implies multivessel grafting through a limited left anterior thoracotomy (86, 87). A further development of this approach is the robotic total endoscopic coronary artery bypass (TECAB) technique which provides multivessel revascularization without an open incision through port access (86). Aim of these less invasive approaches is to reduce the post-operative complications, reduce the surgical trauma and accelerate discharge timing (88).

### 5.2. Aortic valve surgery

Aortic valve (AV) surgery has evolved towards less invasive approaches including mini-sternotomy or right thoracotomy (89). Several studies have shown that patients undergoing less invasive AV surgery have a shorter hospital stay, less pain, shorter duration of ventilation, less blood loss, and less blood transfusion than patients undergoing conventional full sternotomy (90). Postoperatively, patients can be mobilized earlier, and the respiratory function may be better, making this approach particularly suitable for fragile and elderly patients (91).

Less invasive surgical approaches can be implemented with the use of sutureless or rapid deployment valve bio-prostheses. By avoiding placement and tying of sutures after annular decalcification, the use of these valves minimizes cross-clamp and cardiopulmonary bypass times, reduce post-operative morbidity and mortality and improve cost-effectiveness, particularly in high-risk patients as well as in those undergoing complex or concomitant procedures (92–94). Sutureless or rapid deployment aortic valves should be considered for isolated AV replacement in patients with comorbidities, old age, small aortic annulus, delicate aortic wall and conditions such as calcified root (95).

### 5.3. Mitral valve surgery

Similarly to AV surgery, mitral valve (MV) interventions can be performed with techniques to reduce the surgical stress. Right mini-thoracotomy has become the preferred approach for MV surgery at many institutions but it might be burdened by perioperative stroke. Previous cardiac surgery and the severity of aortic and ileo-femoral arterial disease should guide the choice toward an antegrade arterial flow when indicated, and the optimal technique of aortic occlusion and myocardial protection to reduce neurological events (96).

Primary MV regurgitation can also be addressed through off-pump techniques with trans-apical access to the left ventricle through left thoracotomy. This approach is used for the implantation of NeoChord. These artificial chordae tendinae are inserted in the left ventricle, tensioned under echocardiographic guidance, and secured to the left ventricular epicardium using Teflon pledgets (97). This technique has proven effective also in reinterventions and high-risk patients (98). Catheter-based trans-apical mitral valve prosthesis implantation is a potential therapeutic option in high-risk patients but its effect in reducing post-operative morbidity and mortality in frail patients still needs to be demonstrated (99, 100).

### 5.4. Tricuspid valve surgery, right heart failure and atrial fibrillation

MV diseases might be associated with tricuspid valve (TV) regurgitation, pulmonary hypertension, right heart failure and atrial fibrillation (AF). Even though TV disease and AF can be indications for isolated surgical procedures they are often associated with MV surgery in frail patients, increasing the surgical complexity and the risk of postoperative complications. Less invasive approaches through a right mini-thoracotomy and beating heart techniques have been described to address both TV surgery (101, 102) and AF ablation (102). Right mini-thoracotomy has proven to be safe and feasible even in the presence of pulmonary hypertension (103) but its postulated protective role in case of right heart failure is still under discussion.

### 5.5. Ascending aorta and arch surgery

Surgical complexity reaches high levels in case of ascending aorta and aortic arch surgeries where prolonged cardiopulmonary bypass and circulatory arrest are required with potential detrimental effects in frail patients. Moreover, up to 25% of aortic patients fall into the frailty definition (104). Also in this case, less invasive approaches through mini-sternotomy (105) and beating heart techniques to reduce the myocardial ischemic time (106) have been described. Besides the open repair techniques, hybrid arch repair combining vascular and endovascular treatment has gained popularity (107). This approach implies the endovascular exclusion of the pathologic aortic segments following the creation of an adequate proximal landing zone (in zones 0, 1 and 2) by means of supra-aortic transposition (debranching) of one or more arch vessels (107). Finally, a total endovascular aortic arch repair has become possible with the introduction of fenestrated and branched arch endografts (107).

### 5.6. Heart failure surgery

Many heart failure patients fall into the group of frail patients due to their catabolic state, end-organ damage and comorbidities (108). Some of them are candidates for left ventricular assist device (LVAD) implantation but they might suffer from longer time to extubation, longer hospital length of stay, and increased long-term mortality compared to non-frail patients (108). Among the options to reduce their surgical stress, less invasive strategies to implant LVADs have been developed (109). A left anterolateral thoracotomy for pump implant and a mini-sternotomy or a right anterior thoracotomy for the outflow graft anastomosis can be used to implant an LVAD (109, 110), exchange a pump (111) or explant a pump after myocardial recovery (112).

### 5.7. Other intraoperative strategy that could improve outcomes

Literature and clinical practices are lacking specific anesthesiology approaches for frail patients (47, 113). Nevertheless, geriatric principles of anesthesia management can be applied to frail patients (113). This implies the use of lung protective strategies and the reduction of medications potentially inappropriate for older adults, such as long-acting benzodiazepines, diphenhydramine, scopolamine, and promethazine (114). Temperature management during cardiopulmonary bypass should avoid an excessively fast rewarming phase which has been associated with postoperative neurological complications (113). The use of intraoperative anesthesia depth monitoring has been advocated to decrease the amount of medications required and therefore allow for more hemodynamic stability and reduced postoperative delirium and 30-day mortality. Nevertheless, evidence supporting the routine use of electroencephalography-guided anesthesia protocols are still lacking (115).

## 6. Outcome evaluation in frail patients

### 6.1. Postoperative delirium and cognitive impairment

Delirium is a severe neuropsychiatric syndrome characterized by an acute disorder of cognition (mainly but not exclusively attention and awareness), that develops over a short period of time (usually hours to a few days) and represents a change from baseline attention and awareness (116). Delirium tends to fluctuate in severity during the day, and it is almost always caused by underlying medical issues. Risk factors for delirium include age, pre-existing cognitive impairment and dementia, multimorbidity, depression, other psychiatric illnesses, alcohol consumption, poor nutritional status, visual and auditory impairments and frailty (9). The relationship between delirium and frailty is particularly intriguing as these two conditions share similar underlying pathophysiological mechanisms and act as predisposing factors for each other (117, 118). Delirium, in fact, arises from an interplay between predisposing and precipitating factors (9). According to this view, delirium can thus be regarded as a clinical consequence of frailty in older persons experiencing stressful events (118). At least three psychomotor subtypes of delirium can be distinguished: hyperactive (characterized primarily by agitation), hypoactive (characterized mainly by lethargy and drowsiness), and mixed (fluctuation between hyperactive and hypoactive subtypes) (119).

Delirium is a common yet neglected complication after cardiac surgery, affecting about 25%–50% of all patients (120–122). Postoperative delirium (POD) usually occurs within the first four days after surgery (123) and is associated with increased rates of intubation (124) and longer length of hospital stays (121). Delirium has been described by patients as something that affects their emotions and interactions with others and by caregivers as a frightening experience (125). Patients who experience post-operative POD also face an elevated risk of mortality at 30 days and six months after surgery (126, 127), with patients experiencing the hypoactive subtype carrying the worst prognosis (119).

Notably, delirium is also associated with subsequent decline of cognitive functions and risk of dementia (127–129), significantly affecting both individual's and family QoL, and determining increased costs for the society and healthcare systems. Several studies have prospectively assessed cognitive states in medical patients before, during and after delirium, finding a prospective association of delirium with cognitive decline over 2 years and incident dementia (128, 130, 131). A prospective cohort study examined the patterns and pace of cognitive decline during a period of 72 months in 560 community-dwelling older adults who underwent major elective surgery and developed POD. This study demonstrated that patients experiencing POD showed accelerated cognitive decline in comparison to those who either did not develop delirium or did not undergo surgery (128). Globally, these studies demonstrate that delirium is associated with significant cognitive decline in the medium- and long-term.

The mechanisms through which cognitive deficits can develop because of delirium are not entirely understood. However, Davis and colleagues have shown that the pathophysiological mechanisms that contribute to accelerating the progression of cognitive deficits following delirium differ from those implicated in the pathogenesis of dementia (particularly Alzheimer's) which may act independently and additively to the classical pathological processes of dementia (132).

### 6.2. Early recognition, prevention and treatment of postoperative delirium

The initial step for an effective POD treatment is its early recognition. Terms like “acute confusional state,” “toxic-metabolic encephalopathy,” or “psychomotor agitation” should be avoided, while “suspected delirium” should be used for suggestive symptoms (133). However, a proactive strategy to detect the first signs and symptoms of POD is advised. All patients undergoing major surgery (9) should be screened for POD during the first three days after the operation and until resolution of the clinical situation. For this purpose, validated scales such as the 4AT (134) and CAM-ICU (135) scales could be used.

Besides early recognition, it is necessary to reduce precipitating factors that can trigger the onset of POD. A systematic review including 315 articles and 101,144 patients identified 112 precipitating factors associated with the onset of delirium. These factors can be divided into 8 main categories based on pathophysiology: surgical factors, systemic illness or organ dysfunction, metabolic abnormalities, drugs, iatrogenic and environmental factors, trauma, biomarkers, and neurotransmitters (9). Such a complex list of precipitating factors indicates how addressing POD requires a multimodal approach and the contribution of several medical specialists as well as nurses, physiotherapists, psychologists, and other healthcare professionals. Indeed, the currently best approach to POD is non-pharmacological, multicomponent, and interdisciplinary (136–138). The Hospital Elder Life Program (HELP) exemplifies this approach, including interventions like spatial-temporal reorientation (e.g., the use of calendars), limited use of psychoactive drugs, early mobilization, proper sleep hygiene, hydration, nutrition, and sensory aids (139, 140). This program has been shown to be effective in preventing delirium in both medical and surgical patients, potentially reducing cases by 40% (138, 141, 142). Notwithstanding, application of all these measures might be complex and healthcare professionals might experience difficulties in adopting these approaches, often due to staff shortages and different routines (143).

Even when all the above mentioned measures are correctly implemented, POD may still occur. In this case, clinicians should pay attention to POD aetiological treatment (144). All precipitating factors and potential medical causes underlying delirium should be excluded and/or addressed (9). The acronym DELIRIUM, which stands for Drugs, Electrolyte disturbances, Low Oxygen, Infection, Restraints or Reduced sensory input, Intracranial disorders, Urinary and fecal retention, Myocardial and pulmonary disorders (ischemia, heart failure, hypoxia) may aid remembering the most common medical causes of POD (145).

When a patient develop POD, simultaneous supportive care, complications prevention, and behavioral symptoms management are also required (133). Cognitive stimulation and clear communication with patients are essential alongside with family involvement, mobilization, reduction of restraint and devices (bladder catheter, venous access), sensory deficits mitigation (adequate lighting, glasses, hearing aids), and noise reduction (146).

When delirium is severe, causing significant distress to the patient and/or endangering the continuation of life-saving treatments, pharmacological interventions might be necessary (146). It is recommended to start with a single medication at a low dosage (133, 146). The first-line choice is usually haloperidol (0.5/1 mg i.m., repeatable up to a maximum of 5 mg/day) with avoidance of medications with heavy anticholinergic burden (e.g., promazine, chlorpromazine, promethazine) (147). The use of benzodiazepines should be considered only in selected cases, such as Lewy body dementia (marked sensitivity to antipsychotics) and alcohol withdrawal forms (133). Physical restraint should not be routinely used, and it might be considered only if other non-pharmacological and pharmacological approaches have shown to be ineffective (133, 146). In case of physical restraint, monitoring of agitation should be provided regularly to check if restraint measures can be removed (133).

The approach described above underscores the need for comprehensive and coordinated care, emphasizing that managing such a complex syndrome requires a multidisciplinary team dedicated to delirium prevention and management.

### 6.3. Quality of life and functional recovery

Frailty is synonymous with diminished QoL, and its exacerbation by adverse events significantly compounds the distress in these patient cohorts (148). Cardiac surgery aims to enhance QoL and ameliorate patients' prognosis. However, the latter wanes in significance as age advances. Indeed, for older individuals, the preservation of cognitive and functional capacities, along with the sustenance of a high QoL, usually outwits mere life extension (149). While preoperative risk calculators are useful for predicting the risk of mortality in cardiac operations (150), scant guidance is available to anticipate the potential improvement in postoperative QoL. A systematic review conducted in 2015 showed that QoL tends to improve in most octogenarians following cardiac surgery (151). Nonetheless, 8%–19% of them experience a deterioration of QoL, subsequently regretting their decision to proceed with heart surgery. Given the burgeoning population of geriatric patients, identifying those who will not benefit from an improvement of their QoL is pivotal. Therefore, the development of predictive models for postoperative QoL is warranted to improve the quality of informed consent and ameliorate resources allocation (151).

QoL is closely related to the patient's functional status (152) and functional recovery in the postoperative course (153). Notably, functional decline is closely associated with prolonged length of hospital stay, greater use of healthcare resources, increased likelihood of long-term care admission and high mortality risk (154). Pre-operative impairments in ADLs and disability should be assessed to screen those patients who might benefit from post-operative strategizing, especially concerning aid with self-care during the first 4–6 weeks following cardiac surgery (155). It has been demonstrated that >20% of those aged 70 years and above experience functional decline 3 months post hospital admission in comparison to their preadmission functional status (156), with delirium influencing the association between frailty and variation in the IADL score at 1-month (157). This post-hospitalization functional decline could be predicted using a four-variable model at a threshold of ≥1. The contributing factors encompass preadmission daily reliance on assistance in IADL (1 point), use of a walking device (2 point), dependence on assistance for travel (1 point) and no education beyond age 14 (1 point) (156).

In summary, pre-operative assessment of functional and cognitive status might significantly impact care in the post-operative trajectories for frail adults. Further research is warranted to elucidate the role of this potentially powerful tool into routine clinical practice.

## 7. Conclusions

**Figure 2:**
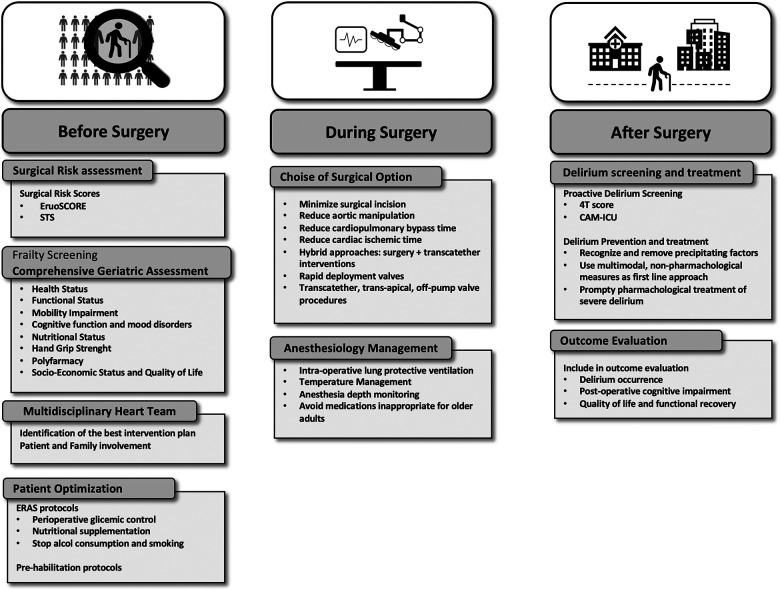
Suggested pathways for pre-operative assessment, introperative optimization and post-operative outcome improvement for frail patients.

Frail individuals are characterized by an increased vulnerability to surgical stress due to the decline of their physiological reserve. Indeed, they are at increased risk for complications and poor outcomes after cardiac surgery. Pre-operative assessment of these patients should incorporate a multidimensional frailty evaluation by CGA. An accurate quantification of surgical risk is the first step to identify vulnerable patients suitable for pre-operative optimization programs. Moreover, it allows a shared decision making process which involves patients and family to ensure a patient-centered definition of goals of care and avoid treatment futility. Patients who are deemed suitable for surgery may benefit from a tailored intraoperative strategy aimed to minimize surgical invasiveness e to enhance postoperative recovery. Finally, an early recognition of possible postoperative complications, such as delirium, may enhance patients' recovery toward a better postoperative quality of life (Figure 2).
